# Multi-Modal Neurorehabilitation for Persisting Post-Concussion Symptoms

**DOI:** 10.1089/neur.2022.0081

**Published:** 2023-05-12

**Authors:** Edward A. Ross, Robert B. Hines, Michael Hoffmann, Kenneth Jay, Matthew M. Antonucci

**Affiliations:** ^1^ Department of Medicine, University of Central Florida, Orlando, Florida, USA.; ^2^ Department of Population Health Sciences, University of Central Florida, Orlando, Florida, USA.; ^3^ Neurology Section, Orlando VA Medical Center, Orlando, Florida, USA.; ^4^ Department of Neurology, Carrick Institute, Cape Canaveral, Florida, USA.; ^5^ Department of Research, Cervello, A/S, Roskilde, Denmark.

**Keywords:** cognitive assessment, concussion, mTBI, neuroplasticity, neurorehabilitation, persisting post-concussive symptoms, post-concussive syndrome, traumatic brain injury

## Abstract

Treatment of the chronic sequela that persist after a mild traumatic brain injury has been challenging with limited efficacy. The aim of this work was to report outcomes obtained from persons who met the criteria of persisting post-concussive symptoms (PPCS), utilizing a novel combination of modalities in a structured neurorehabilitation program. This work was designed as a retrospective, pre-post chart review of objective and subjective measures collected from 62 outpatients with PPCS a mean of 2.2 years post-injury, before and after a multi-modal 5-day treatment protocol. The subjective outcome measure was the 27-item modified Graded Symptom Checklist (mGSC). Objective outcome measures were motor speed/reaction time, coordination, cognitive processing, visual acuity, and vestibular function. Interventions included non-invasive neuromodulation, neuromuscular re-education exercises, gaze stabilization exercises, orthoptic exercises, cognitive training, therapeutic exercises, and single/multi-axis rotation therapy. Pre-post differences in measures were analyzed using the Wilcoxon signed-rank test, with effect size determined by the rank-biserial correlation coefficient. Pre-post treatment comparisons for the subjective mGSC overall, combined symptom measures, individual components of the mGSC, and cluster scores significantly improved for all items. Moderate strength relationships were observed for the mGSC composite score, number of symptoms, average symptom score, feeling in a “fog,” “don't feel right,” irritability, and physical, cognitive, and affective cluster scores. Objective symptom assessment significantly improved for trail making, processing speed, reaction time, visual acuity, and Standardized Assessment of Concussion. Patients suffering from PPCS ∼2 years after injury may have significant benefits with some moderate effect sizes from an intensive, multi-modal neurorehabilitation program.

## Introduction

Treatment of symptoms that persist beyond 3 months after mild traumatic brain injury m(TBI) is an ongoing topic of investigation across populations with heterogeneous traumatic events (e.g., civilian, military, and sports related). Although there has been progress, there is still no consensus on the measures that establish a definitive diagnosis of persisting post-concussive symptoms (PPCS) in any age group. PPCS, including three or more symptoms, occurs between 24% and 43% of those who sustain a concussion^1^ and has been described as permanent when lasting >3 years.^1^ In the acute stage of concussion, the brain experiences widespread neuroinflammation, cellular energy imbalance, and axonal damage to varying degrees.

As a consequence of these processes, neural hub disruption, rerouting, and neuroplasticity/rerouting/reorganization occur. This leads to impaired neural transmission, compromised neurological integrity, and disruption of the local and global processing network.^2^ This mechanism, combined with the individuality of each brain, may explain the heterogenous presentation of PPCS, including constellations of vestibular, visual, emotional, cognitive, sleep, autonomic, and cervical symptoms. The heterogeneous presentation of concussion may also explain why unimodal, protocol-driven approaches may not produce desired outcomes. Though there is some uncertainty whether the chronic PPCS population can benefit from neurorehabilitation^3^ due to a paucity of well-designed, blinded, randomized, and controlled studies, a pilot study,^4^ multiple case-study abstracts,^5,6^ and at least one case-series abstract^7^ have reported encouraging benefits from a multi-method therapeutic approach for subjects at >1 year post-trauma. This was the impetus for this retrospective investigation encompassing a larger number of patients, broader scope of mixed-modality outpatient neurorehabilitation therapies, and more extensive testing measures for symptom severity and cognitive and vestibular function.

## Methods

This was a descriptive, retrospective, single-arm, pre-post study by chart review with patient consent, approved by the University of Central Florida Institutional Review Board (Orlando, FL). Over 24 contiguous months, 418 patients with a confirmed diagnosis of concussion were treated at a tertiary neurorehabilitation center. Specific referral demographics were not available in many of the patient records, but of those that were available, the majority of patients were self-referred (Google search, social media, or word-of-mouth), with a minority being referred by their primary care provider. Inclusion criteria were: 1) a history of a blow to the head, neck, or elsewhere on the body that resulted in a reported neurological sign; at least two symptoms and an examination finding; or an established diagnosis of concussion; 2) symptom duration of ≥3 months; and 3) persisting symptoms after a past rehabilitation intervention. Exclusion criteria were: 1) patients under the age of 13; 2) involved in active litigation; 3) other primary established or comorbid diagnosis (e.g., attention-deficit/hyperactivity disorder, migraine, bipolar disorder, or conversion disorder); 4) failure to perform one or more components of an assessment; 5) break in continuity of treatment >2 days; and 6) incomplete data set.

After applying these criteria, 62 (14.8%) participants met the inclusion criteria, and they are the basis of this report. Of those who were excluded, 27 (7.5%) were under the age of 13, 19 (5.3%) were in active litigation, 192 (53.9%) had pre-morbid/comorbid conditions, 4 (1.1%) failed to perform part(s) of the assessment, 2 had a break in continuity of care (0.6%), and 112 (31.4%) did not have a complete data set (defined as one or more missing values).

### Aim/objective of study

The primary objective was to ascertain whether there was a significant and substantial change of self-reported symptoms and quantitative measures after the 5-day outpatient multi-modal, individualized, and focused therapeutic protocol.

### Assessment methodology

The instrument utilized to record both subjective and objective outcome assessments was the C3Logix (Neurologix Technology, Inc., Cleveland, OH) Apple iPad (Apple, Cupertino, CA) application.^8^

#### Subjective measures

To assess subjective complaints, C3Logix utilizes a modified Graded Symptom Checklist (mGSC) to track patient-reported symptoms, pre- and post-interventions,^9^ and has been particularly useful in assessing cognitive and oculomotor function and postural stability in concussion patients.^8^ The mGCS is a variation of the Post-Concussion Symptom Survey, which consists of 22 concussion-related symptoms, following international recommendations.^10^ Five extra questions were added by C3Logix during development to disaggregate components of a subject's symptomology, yielding the current mGSC. The 27 symptoms in the mGSC are rated by the patient from 0 (“None”) to 6 (“Severe”).^11^ Overall mGSC composite score was obtained by summing values for the symptom scores on the 27 items. Number of symptoms was defined as summing the number of symptoms for each of the 27 items where the symptom assessment was ≥1. Average symptom score was defined as the mGSC composite score divided by the number of symptoms.

Component symptoms were also grouped^12^ into categories of physical, cognitive, sleep, and affective/emotional symptom clusters. C3Logix software provides a symptom count (of 27) and a severity composite score (of 162 possible points) clustered into three groups: 1–54 is mild, 55–109 moderate, and 110–162 severe.

#### Objective measures

The software performs 12 calculations in the domains of motor speed/reaction time, balance, coordination, cognitive processing, visual acuity, and vestibular function. These objective performance metrics (“sensor-based data”) are widely used, well-established neurocognitive metrics that have demonstrated good convergent validity and 1-week test-retest reliability.^9,11^ Pre-established clinical operations facilitate unbiased data acquisition: Assessment provider is never the examining/prescriber provider; treating provider is not the post-assessment provider; all providers are scripted and trained to administer each assessment uniformly; subjective evaluations are performed after all other diagnostic assessments described below; and assessments are scheduled at approximately the same time (±2 h from the intake assessment time).

Performance assessments were: Trail Making Test Parts A and B (TMT-A, TMT-B); processing speed (Symbol Digit Matching; SDM); the Standardized Assessment of Concussion (SAC) with four components of Immediate Memory Recall (IMR), Delayed Memory Recall (DMR), Orientation Assessment (OA), and concentration by Reverse Digit Span (RDS); Simple and Choice Reaction Time (SRT/CRT) assessments of visual-motor reaction time; and Static (SVA) and Dynamic (DVA) Visual Acuity, as well as the difference between the two. The structure, format, and sequence of the assessments were the same for all patients, pre- and post-treatment: mGSC; SAC (OA, IMR, RDS, and DMR); TMT-A; TMT-B; SDM; SRT; CRT; SVA; and DVA.

### Intervention methodology

Patients were interviewed and evaluated by a licensed clinician to compile a comprehensive history and physical examination. Diagnostic testing was performed using diagnostic equipment (Table 1), including: C3Logix, orthostatic vital signs, computerized dynamic posturography,^13,14^ video oculography (testing spontaneous nystagmus, assessment of gaze-holding, horizontal and vertical smooth pursuits, horizontal and vertical saccades, optokinetic responses, and head impulse testing),^13^ monocular and binocular static visual acuity, stereoacuity, eye dominance, color deficiency, contrast sensitivity, accommodation, convergence, divergence, assessment of hand-eye reaction time assessment, and simple and complex tandem gait analysis. The provider customized a rehabilitation plan implementing the following rehabilitation and training protocols.

**Table 1.** The Relative Distribution (Percentage) of the Different Intervention Therapies Applied to the Cohort, and Instrumentation Utilized in the Protocols^1-6^

Distribution of Intervention type		
Intervention type	No. of patients who received intervention	Percentage (%) of cohort (*n* = 62)
Non invasive neuromodulation		
NINM_Trigeminal R	54	87%
NINM_ Trigeminal L	57	92%
NINM_Median R	15	24%
NINM_Median L	1	2%
NINM_Erb's Point R	7	11%
NINM_Erb's Point L	5	8%
NINM_Tibial R	7	11%
NINM_Tibial L	4	6%
NINM_Gait	2	3%
NINM_Radial R	1	2%
NINM_Brachiradialis R	1	2%
Active choreiform		
Figure 8 _R	33	53%
Figure 8_L	15	24%
Gaze stability		
GS_NNYY	45	73%
GS_NN Only	14	23%
Vestibular rehabilitation		
VR_ Halmagyi R	15	24%
VR_ Halmagyi L	3	5%
Orthoptic stimulus		
Orthoptic_ Bead String	22	35%
Orthoptic_Gaze Shifting	2	3%
Gait training	4	6%
Translational exercises	6	10%
Dynavision^a^		
D2_Right Hand	16	26%
D2_Left Hand	6	10%
D2_Both Hands	19	31%
Stroop	7	11%
Neurosensory integration^b^		
NSI_Rotator CCW	1	2%
NSI_T-scope	14	23%
Interactive metronome^c^		
IM_BL Sides	33	53%
IM_R Side	6	10%
IM_L Side	3	5%
PEAK	23	37%
Gaze shifting		
Brain Exercises_L	26	42%
Brain Exercises_R	8	13%
Motion guidance^d^	2	3%
Multi-axis rotational chair^e^		
MARC_Yaw_R	10	16%
MARC_Yaw_L	5	8%
MARC_Ant Pitch	1	2%
MARC_R Yaw_Ant Pitch	18	29%
MARC_L Yaw_Ant Pitch	6	10%
MARC_R Yaw_Post Pitch	32	52%
MARC_L Yaw_Post Pitch	8	13%
MARC_R Roll	19	31%
MARC_L Roll	6	10%
Tilt table	4	6%
Balance training (CAPS targeting)^f^	4	6%

^a^ DynaVision D2 (Dynavision International LLC, West Chester, OH).

^b^ Neuro Sensorimotor Integrator (RKB Instruments, Gold Canyon, AZ).

^c^ IM Rehab Package (Interactive Metronome, Inc, Sunrise FL).

^d^ Motion Guidance Rechargeable Professional Clinician Kit (Motion Guidance LLC, Denver, CO).

^e^ MARC (ReNeuro Robotics, Inc., Dallas, TX).

^f^ CAPS^®^ Professional Force plate (Vestibular Technologies, LLC, Cheyenne, WY).

Abbreviations: NINM_Trigeminal R, Non Invasive Neuromodulation on the Right Trigeminal Nerve; NINM_ Trigeminal L, Non Invasive Neuromodulation on the Left Trigeminal Nerv; Non Invasive Neuromodulation on the Right Median Nerve; NINM_Median L, Non Invasive Neuromodulation on the Left Median Nerve; NINM_Erb's Point R, Non Invasive Neuromodulation on the Right Erb's Point (Brachial Plexus); NINM_Erb's Point L, Non Invasive Neuromodulation on the Left Erb's Point (Brachial Plexus); NINM_Tibial R, Non Invasive Neuromodulation on the Right Tibial Nerve; NINM_Tibial L, Non Invasive Neuromodulation on the Left Tibial Nerve; NINM Gait, Non Invasive Neuromodulation stimulating nerves involved with gait; NINM_Radial R, Non Invasive Neuromodulation on the Right Radial Nerve; NINM Brachioradialis R, Non Invasive Neuromodulation on the belly of the Right Brachioradialis Muscle; Figure 8 _R, Active choreiform, figure-of-8 movements of the right upper and lower extremities; Figure 8_L, Active choreiform, figure-of-8 movements of the upper and lower extremities; GS_NNYY, Sinusoidal Times-one Gaze Stability around the head z-axis (No-no) and y-axis (Yes-yes); GS_NN Only, Sinusoidal Times-one Gaze Stability around the head z-axis (No-no); GS_YY Only, Sinusoidal Times-one Gaze Stability around the head y-axis (yes-yes); VR_ Halmagyi R, Vestibular Rehab Right Z-axis Head Thrusts; VR_ Halmagyi L, Vestibular Rehab Left Z-axis Head Thrusts; Orthoptic_ Bead String, Orthoptic exercises convergent and divergent saccades using a Brock string; Orthoptic_Gaze Shifting, Orthoptic exercises convergent and divergent saccades using near and far targets; Gait Training, Neuromuscular re-education exercises to promote proper gait patterns; Translational Exercises, Moving a patient towards and away from a target; D2_Right Hand, Dynavision D2 hand-eye visuomotor training system using right hand only; D2_Left Hand, Dynavision D2 hand-eye visuomotor training system using left hand only; D2_Both Hands, Dynavision D2 hand-eye visuomotor training system using both hand; NSI_Rotator CCW, NeuroSensory Integrator hand-eye-cognitive training system with counter-clockwise rotating targets; NSI_T-scope, NeuroSensory Integrator hand-eye-cognitive system tachistoscope; IM_BL Sides, Interactive Metronome sensory-motor entrainment system utilizing bilateral arms and/or legs; IM_R Side, Interactive Metronome sensory-motor entrainment system utilizing right arm/leg; IM_L Side, Interactive Metronome sensory-motor entrainment system utilizing left arm/leg; PEAK Brain Exercises, Cognitive Training Exercises using Peak-Brain Training for iOS (Brainbow Ltd, London, UK); Brain Exercises R, Gaze shifting exercises in a 45° up left/down right pattern developed by FR Carrick (www.CarrickInstitute.com) Brain Exercises L, Gaze shifting exercises in a 45° up right/down left pattern developed by FR Carrick (www.CarrickInstitute.com); Motion Guidance, Laser-guided visual feedback exercises (Motion Guidance, Colorado, USA); MARC_Yaw_R, Whole-Body Rotation clockwise yaw rotation (MARC: Multi-Axis Rotational Chair, ReNeuro Robotics, Texas, USA); MARC_Yaw_L, Whole-Body Rotation counter-clockwise yaw; MARC_Ant Pitch, Whole-Body Rotation positive pitch rotation; MARC_R Yaw_Ant Pitch, Multi-axis whole body rotation with a combination of clockwise yaw rotation and anterior pitch; MARC_L Yaw_Ant Pitch, Multi-axis whole body rotation with a combination of counter-clockwise yaw and anterior pitch; MARC_R Yaw_Post Pitch, Multi-axis whole body rotation with a combination of clockwise yaw and posterior pitch; MARC_L Yaw_Post Pitch, Multi-axis whole body rotation with a combination of counter-clockwise yaw and posterior pitch; MARC_R Roll, Whole-body rotation clockwise roll; MARC_L Roll, Whole-body rotation counter-clockwise roll; Tilt Table, Progressive inclination from 0° (horizontal) to 90° (vertical); Balance Training (CAPS Targeting), Weight-shifting exercise using BalancTrack software (Vestibular Technologies, Wyoming, USA)

#### Non-invasive neuromodulation

Electric nerve stimulation (non-invasive neuromodulation; NINM) was performed by stimulation of the median and trigeminal nerves^15^ and uniformly delivered with asymmetric square wave pulses of 0.1-ms duration, at a frequency of 3 Hz, with increasing intensity from 1 to 20 mA until a muscle fasciculation response was observed. The stimulus was delivered for a total of 30 sec and repeated three times per session.

#### Therapeutic exercises and neuromuscular re-education

Disruption in motor performance and control is a sequela of concussion.^16^ A combination of therapeutic exercises (facilitating motor function) and neuromuscular re-education (promoting cognitive awareness of motor function) was performed, including: rhythmic movement exercises synchronized to a metronome, sensory-motor strategies involving gait patterning exercises, marching exercises using exaggerated limb movements, laser-guided orchestrated limb, trunk, and neck movements in various patterns (passive and active) and visuomotor reaction speed tasks.

#### Gaze stabilization exercises

Vestibular rehabilitation strategies often include gaze stability exercises to strengthen the vestibular-ocular reflex under both dynamic conditions (e.g., gait), in static balance conditions,^17,18^ and these exercises incorporated sinusoidal, passive, horizontal and vertical, dynamic head movements while the patient's eyes were fixated on a stationary target.

#### Cognitive training

Cognitive post-concussion rehabilitation^19^ interventions included: exercises using recall of sequences of letters or numbers; Stroop exercises; dual-tasking; spatial processing; tachistoscope- and cognitive-based games from different applications (PEAK Brain Training [PopReach Corporation, Toronto, Ontario, Canada], Stroop Effect [Attila Hegedus, Italy], and Recognize [Neuro Orthopaedic Institute (Australasia) Pty, Ltd. Adelaide, Australia]) performed on an iPad.

#### Single/multi-axis rotation therapy

Whole-body rotation has proven to be effective in improving stability and reducing sway.^20^ Rotational therapy was performed using a multi-axis rotational chair. Movements were in one or two planes, including yaw, pitch, and roll, at speeds up to 90 degrees per second, with acceleration/deceleration parameters that matched Sadeghi and colleagues,^21^ in three sets of ∼30 sec per session.

#### Orthostatic challenge exercises

If a patient had postural autonomic dysfunction, our protocol included orthostatic challenge exercises in the form of tilt-table therapy^22^ or combined the use of supine, seated, side-lying, and standing progressions during the neuromuscular re-education and therapeutic exercises.

#### General program

Each patient was treated individually using a combination of the above-mentioned treatment options (e.g., neuromodulation therapies prescribed in >90% and gait training needed in <10%), and the specific interventions are shown in Table 1. The rehabilitation was administered in a 12-session, 5-day, contiguous, progressive, outpatient program. Each session was ∼1 h in duration. Patients were instructed to physically and cognitively rest between sessions. No medication, supplements, or dietary regimens were altered during the treatment period.

### Statistical analysis

Bivariate descriptive statistics for PPCS patients are presented as medians with interquartile range (IQR), according to pre-post intervention measurement. Outcome measures were changes in ordinal measures pre-post treatment assessed by the Wilcoxon signed-rank test. Effect sizes were obtained by the rank-biserial correlation coefficient and interpreted as the absolute value of the correlation coefficient as follows: very weak (0.00–0.19); weak (0.20–0.39); moderate (0.40–0.59); strong (0.60–0.79); and very strong (0.80–1.00). All measures of assessment were defined *a priori* and are reported.

As recommended by a number of statistical experts, we chose not to adjust *p* values for multiple comparisons.^23,24^ Our rationale for this approach is that, first, this was a preliminary study. Second, all study comparisons were planned *a priori*. Third, *p*-value adjustment is overly conservative with popular approaches like the Bonferroni method.^23–25^ Finally, results of the hypothesis tests in this study were consistent across nearly all outcomes.

## Results

The study population consisted of 62 subjects: 24 women and 38 men (38.7% and 61.3%, respectively). Mean time from the most recent brain injury to presentation was 2.2 years (standard deviation [SD] = 1.5), with a range of 3.1 months to 5.3 years. Regarding age, 9 subjects (14.5%) were 13–17 years of age (yoa), 43 (69.4%) 18–49 yoa, and 10 (16.1%) 50–71 yoa. Of additional interest were the comorbid diagnoses of autonomic dysfunction^26^ in 44 (71.0%) and central vestibulopathy^27^ in 60 (96.8%). Subjects completed 4.8 ± 0.9 days (mean ± SD), with a maximum treatment duration of 8 days.

**FIG. 1.**
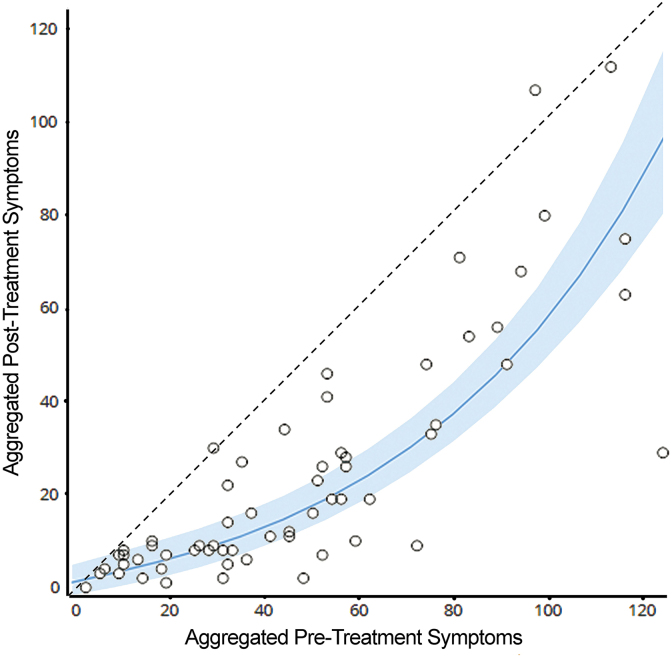
Pre- and post-composite (aggregate) symptom scores (*n* = 62). Shaded curve shows mean with SEM boundaries. SEM, standard error of the mean.

In Table 2, subjective (symptom-based) testing results showed significant improvement in symptom severity based on the composite mGSC score, number of symptoms, average symptom score, and individual symptom components making up the mGSC. Comparing pre-post medians and the rank-serial correlation coefficient, moderate strength relationships were observed for the mGSC composite score (44.5 vs. 13.0, −0.495), number of symptoms (17.5 vs. 10.5, −0.495), average symptom score (2.4 vs. 1.6, −0.484), feeling slowed down (3 vs. 1, −0.430), feeling in a fog (3 vs. 1, −0.447), and irritability (1 vs. 0, −0.423) (Fig. 1).

**Table 2.** Subjective Symptom Assessment of mTBI Patients Before/After Treatment (*n* = 62)

Combined symptoms	Pre-treatment median (IQR)	Post-treatment median (IQR)	*p* value	Rank-biserial correlation	Effect-size interpretation
mGSC composite score	44.5 (25–62)	13 (7–33)	<0.001	–0.495	Moderate
No. of symptoms	17.5 (12–22)	10.5 (5–16)	<0.001	–0.495	Moderate
Average symptom score	2.4 (1.9–3.0)	1.6 (1.1–2.3)	<0.001	–0.484	Moderate
Individual symptom components					
Headache	2 (0–3)	0 (0–2)	<0.001	–0.317	Weak
Pressure in head	2 (1–3)	1 (0–2)	<0.001	–0.289	Weak
Neck pain	1 (0–3)	1 (0–2)	0.003	–0.126	Very weak
Nausea or vomiting	0 (0–1)	0 (0–0)	<0.001	–0.209	Weak
Dizziness	1 (0–3)	0 (0–1)	<0.001	–0.310	Weak
Blurred vision	1 (0–2)	0 (0–1)	<0.001	–0.253	Weak
Balance problems	1 (0–2)	0 (0–1)	<0.001	–0.299	Weak
Sensitivity to light	2 (0–3)	1 (0–2)	<0.001	–0.238	Weak
Sensitivity to noise	1 (0–3)	0 (0–1)	<0.001	–0.319	Weak
Feeling slowed down	3 (1–4)	1 (0–2)	<0.001	–0.430	Moderate
Feeling in a fog	3 (1–4)	1 (0–2)	<0.001	–0.447	Moderate
Don't feel right	2 (1–4)	0.5 (0–2)	<0.001	–0.363	Weak
Difficulty concentrating	3 (1–4)	1 (0–2)	<0.001	–0.392	Weak
Difficulty remembering	2 (0–3)	1 (0–2)	<0.001	–0.349	Weak
Fatigue	3 (1–4)	1 (0–3)	<0.001	–0.371	Weak
Confusion	1 (0–2)	0 (0–1)	<0.001	–0.291	Weak
Drowsiness	1.5 (0–3)	0.5 (0–2)	<0.001	–0.264	Weak
Trouble falling asleep	1 (0–3)	0 (0–1)	<0.001	–0.226	Weak
More emotional	1 (0–2)	0 (0–1)	<0.001	–0.278	Weak
Irritability	1 (0–3)	0 (0–1)	<0.001	–0.423	Moderate
Sadness	1 (0–2)	0 (0–0)	<0.001	–0.290	Weak
Nervous or anxious	1 (0–3)	0 (0–1)	<0.001	–0.340	Weak
Sleeping more than usual	0 (0–2)	0 (0–1)	<0.001	–0.198	Very weak
Sleeping less than usual	0 (0–2)	0 (0–0)	<0.001	–0.215	Weak
Difficulty sleeping soundly	1 (0–3)	0 (0–1)	<0.001	–0.185	Very weak
Ringing in the ears	0 (0–2)	0 (0–1)	<0.001	–0.180	Very weak
Numbness or tingling	0 (0–2)	0 (0–0)	<0.001	–0.100	Very weak

mTBI, mild traumatic brain injury; mGSC, modified Graded Symptom Checklist; IQR, interquartile range.

**FIG. 2.**
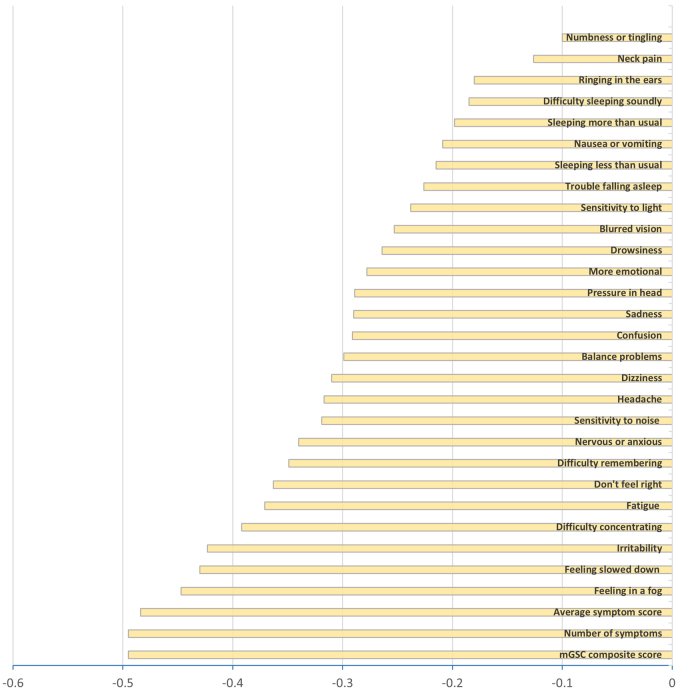
Rank-biserial correlations* for effect size for symptom improvement. *Designated as very weak (0.00–0.19), weak (0.20–0.39), and moderate (0.40–0.59) effects. mGSC, modified Graded Symptom Checklist.

Table 3 expresses the findings when grouped into symptom clusters, all of which were substantially (*p* < 0.001) better. Comparing pre-post medians and the rank-serial correlation coefficient (Fig. 2), moderate strength relationships were observed for the physical cluster score (14.5 vs. 6.0, −0.418), cognitive cluster score (16 vs. 5, −0.487), and affective cluster score (4.0 vs. 0.5, −0.450).

**Table 3.** Symptom Cluster Scores Before/After Treatment for All Subjects (*n* = 62)

Symptom cluster	Pre-treatment median (IQR)	Post-treatment median (IQR)	*p* value	Rank-biserial correlation	Effect-size interpretation
Physical cluster score	14.5 (7–24)	6 (2–12)	<0.001	–0.418	Moderate
Cognitive cluster score	16 (7–23)	5 (2–13)	<0.001	–0.487	Moderate
Sleep cluster score	6.5 (2–12)	2.5 (1–5)	<0.001	–0.359	Weak
Affective cluster score	4 (1–9)	0.5 (0–4)	<0.001	–0.450	Moderate

IQR, interquartile range.

The results of the objective (sensor-based) testing are shown in Table 4. The number of patients for each analysis is included because some subjects did not complete one or both pre-post assessments and were excluded from the analysis. All comparisons were statistically significant except visual acuity line difference (which was borderline statistically significant) and the SAC subcomponents OA, IMR, and DMR. Effect sizes were smaller in magnitude for the objective measures, with most (9 of 13) being weak.

**Table 4.** Objective Symptom Assessment of mTBI Patients Before/After Treatment

Measures	*n*	Pre-treatment median (IQR)	Post-treatment median (IQR)	*p* value	Rank-biserial correlation	Effect-size interpretation
Trails A	61	22.6 (17.8–29.5)	19.3 (15.2–23.4)	<0.001	–0.232	Weak
Trails B	62	44.5 (38.5–57.1)	37.8 (29.4–47.2)	<0.001	–0.319	Weak
Processing speed	60	56 (51–65)	61 (55–71)	<0.001	0.245	Weak
Simple reaction time	62	320.9 (292.3–352.5)	299.7 (270.1–323.9)	<0.001	–0.305	Weak
Choice reaction time	61	436.8 (387.1–498.8)	401.7 (370.5–448.2)	<0.001	–0.294	Weak
Visual acuity S	62	−0.19 (−0.28 to −0.10)	−0.26 (−0.30 to −0.16)	0.001	–0.206	Weak
Visual acuity D	62	−0.10 (−0.20 to 0.00)	−0.18 (−0.26 to −0.08)	<0.001	–0.253	Weak
Visual acuity line difference	62	0.9 (0.2–1.2)	0.6 (0.2–1.0)	0.066	–0.126	Very weak
SAC	62	28 (26–29)	29 (27–30)	0.001	0.265	Weak
SAC subcomponent: orientation	62	5 (5–5)	5 (5–5)	0.544	0.047	Very weak
SAC subcomponent: concentration	62	4 (3–5)	5 (4–5)	0.019	0.229	Weak
SAC subcomponent: immediate memory recall	62	15 (14–15)	15 (14–15)	0.586	0.074	Very weak
SAC subcomponent: delayed memory recall	62	4 (3–5)	5 (3–5)	0.086	0.112	Very weak

mTBI, mild traumatic brain injury; IQR, interquartile range; SAC, Standardized Assessment of Concussion.

## Discussion

Heterogeneous causes of TBI present challenges for relating pathophysiology to symptoms and optimizing methods to monitor and/or treat patients. As reviewed by Kenzie and colleagues,^2^ a concussion is a blow to the head, neck, or elsewhere on the body, generating impulsive forces to the brain, initiating a neurovascular-inflammatory-metabolic injury that alters neurological function. Often, the terms concussion and mild traumatic brain injury (mTBI) are used synonymously. Though there is a paucity of unified definitions, consensus is building as demonstrated by the American Congress of Rehabilitation Medicine's criteria^28^ for the maximal severity of concussion and mTBI; measures that were used to identify the subjects of this report. Whereas symptoms associated with concussion typically self-resolve in all populations in <20 days, the treatment interventions presented in the present study for PPCS have both similarities and differences to the clinical practice guidelines consensus as described by Marshall and colleagues.^29^ It is evident that the complexity of PPCS is extensive and likely requires an individualized diagnostic-driven rehabilitation strategy. Accordingly, a targeted approach for treatment protocols to address a patient's specific deficits has been previously advocated in the rehabilitation literature.^4,30,31^

In the treatment of acute and subacute concussion, as well as early PPCS, therapeutic modalities that have previously been reported to be of benefit in studies addressing various ranges of injury severity include cognitive rehabilitation,^32^ computer-based cognitive training,^33^ vestibular rehabilitation, vision therapy, task-based rehabilitation,^3^ physical therapy,^34^ robot-assisted repetitive movement, biofeedback, and various psychological interventions,^34^ orthoptic/oculomotor rehabilitation,^35^ vestibular rehabilitation,^36,37^ chiropractic manipulation,^38^ physiotherapy,^39^ aerobic exercise^40^, and NINM.^41^ Rehabilitation sessions are typically spread out over weeks to months and often in isolation from other therapies or in a serial fashion. There are very few studies using the multi-modal outpatient rehabilitation we adopted for PPCS,^3,42,43^ and others have a small sample size.^34,44,45^

Notably, in one of the better-documented approaches to PPCS rehabilitation, Wing and colleagues^46^ proposed a multi-modal and individualized treatment strategy in the rehabilitation of PPCS, including: complex multi-step problem solving; logic puzzles; memory challenges; digital therapeutic games; visual exercises; visuospatial retraining; psychosocial therapy; neuromuscular therapy; and aerobic exercise. More recently, in their pilot study, Kontos and colleagues^4^ reported highly encouraging results for a limited repertoire of multi-modal therapies in a small group of subjects between 1 and 3 years after their injury. Our findings support and extend their preliminary results, showing improvements in some measures of symptoms and memory, balance, and oculomotor function. The results of the present study are most aligned with the retrospective non-controlled study of severe PPCS by Carrick and colleagues,^42^ in which patients received head-eye vestibular motion therapy for an intensive treatment period of 5 days and there was a significant decrease in severity scores with a large effect size of 0.83.

Salaffi and colleagues^47^ reported that, on average, a reduction of 15% represented a minimal clinically important difference for chronic musculoskeletal pain patients measured on a numerical rating scale and a score change −33.0% was best associated with the concept of “much better” improvement. Using the approach described by Lemieux and colleagues^48^ and the observations made by Salaffi and colleagues^47^ and Hurst and Bolton,^49^ the results of the present report overall reflect clinically important, meaningful changes in subjective and objective measures in a PPCS population. The subjective tests, as a composite, were improved by 48.6% (with improvement in individual tests by as much as 86%).

Benefits were demonstrated in all four clusters of symptoms (physical, cognitive, sleep, and affect) and encompassed those typically associated with complicated mTBI (headache, problems with memory and concentration, fatigue, sensitivity to noise and light, dizziness, imbalance or other vestibular symptoms, and emotion complaints). It should be noted that although no psychotherapy or mood-stabilizing drugs were administered, the second largest improvement was observed in the affective/mood symptom domain. Benefits were substantiated by clinically relevant effect sizes (Fig. 2), and statistically significant changes in well-accepted objective measures, that relate to common patient deficits and complaints: trail-making; processing speed; reaction time; and visual testing.

We hypothesize from our observations that neuroplasticity and sensory entrainment created by rigorous, diagnostic- and problem-focused, structured neurorehabilitation can be efficacious in the chronic condition, even in the late phase of PPCS, when patients have clinically plateaued.

The present study has numerous limitations, largely inherent to its design. Being a retrospective chart review, it was exploratory in nature. Limitations include: selection bias (patients voluntarily chose the intervention and paid for it); exploratory outcome measures; retrospective design without a control group (patients may act as a self-control because of a history of plateauing after past therapies); lack of financial analyses (e.g., cost/benefit in light of limited insurance coverage); non-uniform treatments attributable to patient heterogeneity; lack of longitudinal follow-up (e.g., durability of outcomes); the possibility of unknown/undisclosed pre-existent comorbidities (e.g., neurological, medical) and previous treatment protocols; wide and non-normally distributed age range; multiple comparisons on the data set; the highly subjective nature of many measures (e.g., sleep adequacy); and ceiling effects to some assessments (e.g., sensor-based assessments SAC, TMT; SRT; CRT; SVA; and DVA); SAC and mGSC are not designed to be used for PPCS.

In conclusion, using subjective and objective assessment tools, this preliminary study suggests that late-stage PPCS patients with symptoms ∼2 years after their injury may have substantive benefits of a clinically relevant magnitude from a patient-centered, intensive, 5-day, multi-modal, diagnostic, and problem-focused outpatient neurorehabilitation program, utilizing a novel combination of traditional and innovative outpatient protocols technology. Future studies, using rigorous, randomized controlled study designs and methods to test for the neuroplastic remodeling of sensory-cognitive-motor networks, are recommended to confirm these observations.

## Abbreviations Used

CRT: Choice Reaction Time
DMR: Delayed Memory Recall
DVA: Dynamic Visual Acuity
IMR: Immediate Memory Recall
IQR: interquartile range
mGSC: modified Graded Symptom Checklist
mTBI: mild traumatic brain injury
NINM: non-invasive neuromodulation
OA: Orientation Assessment
PPCS: persisting post-concussive symptoms
RDS: Reverse Digit Span
SAC: Standardized Assessment of Concussion
SD: standard deviation
SDM: Symbol Digit Matching
SRT: Simple Reaction Time
SVA: Static Visual Acuity
TBI: traumatic brain injury
TMT-A: Trail Making Test Part A
TMT-B: Trail Making Test Part B
yoa: years of age
